# Subjective Outcome Evaluation of a Positive Youth Development Program in Hong Kong: Profiles and Correlates

**DOI:** 10.1100/tsw.2010.2

**Published:** 2010-02-12

**Authors:** Hing Keung Ma, Daniel T.L. Shek

**Affiliations:** ^1^ Department of Education Studies, Hong Kong Baptist University, Shanghai, China; ^2^ Department of Applied Social Sciences, Hong Kong Polytechnic University, Shanghai, China; ^3^ Department of Sociology, East China Normal University, Shanghai, China; ^4^ Kiang Wu Nursing College of Macau, Macao

**Keywords:** positive youth development program, Hong Kong, subjective outcome evaluation

## Abstract

Secondary school students (n = 33,867 from 213 secondary schools) responded to a subjective outcome evaluation form to assess their views of the program, workers (teachers and/or social workers), and perceived effectiveness of the program. Results showed that high proportions of the respondents had positive perceptions of the program and the instructors, and more than four-fifths of the respondents regarded the program as helpful to them. While schools admitting students with different academic abilities and hours did not differ in the subjective outcome evaluation ratings, subjective evaluation ratings for workers were highest, followed by ratings for the program and perceived effectiveness. The present study replicates the previously reported findings and provides additional support for the effectiveness of the Tier 1 Program of the Project P.A.T.H.S. (Positive Adolescent Training through Holistic Social Programmes) in Hong Kong.

## INTRODUCTION

With the intensification of adolescent problems in the context of Hong Kong, there is a demand for positive youth development programs for adolescents[1,2,3]. To promote holistic development among adolescents in Hong Kong, The Hong Kong Jockey Club Charities Trust initiated a project entitled "P.A.T.H.S. to Adulthood: A Jockey Club Youth Enhancement Scheme". The word "P.A.T.H.S." denotes Positive Adolescent Training through Holistic Social Programmes. A research team with researchers from five universities in Hong Kong was formed with The Hong Kong Polytechnic University as the lead institution and the second author as the Principal Investigator, in order to develop a multiyear, universal, positive youth development program to promote holistic adolescent development in Hong Kong[4,5]. There are two tiers of programs in the Project P.A.T.H.S.: Tier 1 and Tier 2. The Tier 1 Program is a universal, positive youth development program where students in Secondary 1–3 participate in 10–20 h of training in the school year at each grade, involving 20–40 teaching units. In the Tier 1 Program, 15 positive youth development constructs are included in the 40 teaching units[6].

Subjective outcome evaluation is one of the evaluation mechanisms of the Project P.A.T.H.S. As the Project P.A.T.H.S. was financially supported by The Hong Kong Jockey Club Charities Trust, each participating school was required to submit an evaluation report to the funding body with the consolidated subjective outcome evaluation profile of the school. In other words, as the workers were expected to conduct program evaluation as part of their professional practice, we could make use of such reports to "reconstruct" the overall profile of the subjective outcome evaluation data based on the perspectives of the program participants and program implementers. Several studies have shown that the program participants perceived the program and program implementers positively, and they also identified the benefits of the programs to their psychosocial development[7,8,9,10,11]. Similarly, subjective outcome evaluation based on the perspective of the program implementers revealed that the program implementers had positive perceptions of the program and themselves as the implementers, and they also felt that the program could promote the psychosocial development of the program participants[12,13,14,15]. In short, the subjective outcome evaluation findings based on several studies demonstrate the perceived effectiveness of the program from the perspectives of the participants and program implementers.

This paper intends to present the results of the subjective outcome evaluation of the Tier 1 Program by examining different domains of the subjective outcome evaluation (e.g., perceptions of the program, program implementers [teachers and/or social workers], perceived program effectiveness, and overall satisfaction) based on the views of the program participants. It also examines the possible differences among these subjective outcome evaluation domains. Moreover, it is of interest to examine the differences in these subjective outcome evaluation domains between schools adopting the 10-h core program mode and schools adopting the 20-h full program mode. Furthermore, as students in Hong Kong are categorized in accordance with their academic achievement (band 1: students with high academic achievement; band 2: students with medium academic achievement; band 3: students with low academic achievement), this study also investigates whether these students have different views of the positive youth development program, program workers, and program effectiveness.

## METHODS

### Participants and Procedures

There were 213 schools that joined the Secondary 1 Program of the Project P.A.T.H.S. in the second year of the Full Implementation Phase in the 2007/08 school year. The mean number of students per school was 171.05 (range: 16–267), with an average of 4.69 classes per school (range: 1–8). Among them, 105 schools adopted the full program (i.e., 20-h program involving 40 units), while 108 schools adopted the core program (i.e., 10-h program involving 20 units). The mean number of sessions used to implement the program was 23.61 (range: 5–60). While 116 (54.46%) schools incorporated the program into the formal curriculum (e.g., Liberal Studies, Life Education), 97 schools (45.54%) used other modes (e.g., form teachers' periods) to implement the program. The mean numbers of social workers and teachers implementing the program per school were 2 (range: 0–8) and 5.63 (range: 0–28), respectively.

After the Tier 1 Program was completed, the participants were invited to respond to a subjective outcome evaluation questionnaire. A total of 33,867 students (mean = 159 students per school, range: 14–267) responded to the Subjective Outcome Evaluation Form (Form A) developed by the research team[16]. The data collection was normally carried out at the last session of the program. On the day when the evaluation data were collected, the purpose of the evaluation was mentioned and confidentiality of the data collected was repeatedly emphasized to all of the students. The students were asked to indicate if they did not want to respond to the evaluation questionnaire (i.e., "passive" informed consent was obtained from the students). All participants responded to all scales in the evaluation form in a self-administration format. Adequate time was provided for the participants to complete the questionnaire. To facilitate the program evaluation, the research team developed an evaluation manual with standardized instructions for collecting the subjective outcome evaluation data[16]. In addition, adequate training was provided to the workers during the 20-h training workshops on how to collect and analyze the data collected by Form A.

## Instruments

The Subjective Outcome Evaluation Form (Form A) was designed by Shek and Siu[16]. Broadly speaking, there are several parts in the evaluation form as follows:

- Participants' perceptions of the program, such as program objectives, design, classroom atmosphere, interaction among the students, and the respondents' participation during class (10 items)
- Participants' perceptions of the workers (teachers and/or social workers), such as the preparation of the instructor, professional attitude, involvement, and interaction with the students (10 items)
- Participants' perceptions of the effectiveness of the program, such as promotion of different psychosocial competencies, resilience, and overall personal development (16 items)
- The extent to which the participants would recommend the program to other people with similar needs (one item)
- The extent to which the participants would join similar programs in future (one item)
- Overall satisfaction with the program (one item)
- Things that the participants learned from the program (open-ended question)
- Things that the participants appreciated most (open-ended question)
- Opinion about the instructor(s) (open-ended question)
- Areas that require improvement (open-ended question)

After receiving the consolidated data by the funding body, the data were aggregated to "reconstruct" the overall profile based on the subjective outcome evaluation data by the research team. Besides looking at the percentage scores, several composite scores were also computed. First, the views of the program participants, program implementers, and program effectiveness, in terms of percentages, were computed. Second, three overall ratings (means and standard deviations) were computed: (1) VP: the average of the 10 items regarding the views of the program, (2) WK: the average of the 10 items regarding the views of the workers (including teachers and social workers) implementing the program, and (3) EF: the average of the 16 items regarding the views of the perceived effectiveness of the program.

## RESULTS

Reliability analysis with the schools as the unit of analyses showed that Form A was internally consistent: 10 items related to the program (alpha = 0.98, mean interitem correlation = 0.85), 10 items related to the instructor (alpha = 0.99, mean interitem correlation = 0.94), 16 items related to the benefits (alpha = 0.99, mean interitem correlation = 0.93), and 39 items based on the entire Form A (alpha = 0.99, mean interitem correlation = 0.80).

The quantitative findings based on the closed-ended questions are presented in this paper. There are several observations that can be highlighted from the findings. First, more than two-thirds of the respondents perceived the program in a positive manner (Table 1). For example, 84.97% of the students indicated that the program objectives were very clear; 82.78% felt that the activities were carefully planned. Second, a high proportion of the students had a positive evaluation of the instructors (Table 2). For example, 89.74% of the respondents indicated that the instructors were very involved; 89.13% of the

## TABLE 1
## Summary of the Views of the Program Participants of the Program

| | 1 | | 2 | | 3 | | 4 | | 5 | | 6 | | Participants with Positive Responses (Options 4–6) | |
|---|---|---|---|---|---|---|---|---|---|---|---|---|---|---|
| | Strongly Disagree | | Disagree | | Slightly Disagree | | Slightly Agree | | Agree | | Strongly Agree | | | |
| | n | % | n | % | n | % | n | % | n | % | n | % | n | % |
| The objectives of the curriculum were very clear. (n = 33,567) | 1,033 | 3.08 | 961 | 2.86 | 3,052 | 9.09 | 10,474 | 31.20 | 13,530 | 40.31 | 4,517 | 13.46 | 28,521 | 84.97 |
| The design of the curriculum was very good. (n = 33,553) | 946 | 2.82 | 1,310 | 3.90 | 4,007 | 11.94 | 11,984 | 35.72 | 11,659 | 34.75 | 3,647 | 10.87 | 27,290 | 81.33 |
| The activities were carefully planned. (n = 33,492) | 891 | 2.66 | 1,133 | 3.38 | 3,742 | 11.17 | 11,461 | 34.22 | 12,294 | 36.71 | 3,971 | 11.86 | 27,726 | 82.78 |
| The classroom atmosphere was very pleasant. (n = 33,437) | 1,230 | 3.68 | 1,421 | 4.25 | 3,953 | 11.82 | 9,462 | 28.30 | 11,135 | 33.30 | 6,236 | 18.65 | 26,833 | 80.25 |
| There was much peer interaction among the students. (n = 33,278) | 1,068 | 3.21 | 1,270 | 3.82 | 3,535 | 10.62 | 9,710 | 29.18 | 11,447 | 34.40 | 6,248 | 18.78 | 27,405 | 82.35 |
| I participated actively during lessons (including discussions, sharing, games, etc.). (n = 33,496) | 1,130 | 3.37 | 1,391 | 4.15 | 3,597 | 10.74 | 10,417 | 31.10 | 11,379 | 33.97 | 5,582 | 16.66 | 27,378 | 81.74 |
| I was encouraged to do my best. (n = 33,476) | 1,337 | 3.99 | 1,728 | 5.16 | 4,519 | 13.50 | 11,708 | 34.97 | 10,285 | 30.72 | 3,899 | 11.65 | 25,892 | 77.34 |
| The learning experience I encountered enhanced my interest towards the lessons. (n = 33,368) | 1,303 | 3.90 | 1,706 | 5.11 | 4,286 | 12.84 | 11,287 | 33.83 | 10,734 | 32.17 | 4,052 | 12.14 | 26,073 | 78.14 |
| Overall, I have a very positive evaluation of the program. (n = 33,431) | 1,378 | 4.12 | 1,753 | 5.24 | 4,540 | 13.58 | 10,974 | 32.83 | 10,604 | 31.72 | 4,182 | 12.51 | 25,760 | 77.05 |
| On the whole, I like this curriculum very much. (n = 33,337) | 1,609 | 4.83 | 1,757 | 5.27 | 3,844 | 11.53 | 10,133 | 30.40 | 10,531 | 31.59 | 5,463 | 16.39 | 26,127 | 78.37 |

## TABLE 2
## Summary of the Views of the Program Participants of the Program Implementers

| | 1 | | 2 | | 3 | | 4 | | 5 | | 6 | | Participants with Positive Responses (Options 4–6) | |
|---|---|---|---|---|---|---|---|---|---|---|---|---|---|---|
| | Strongly Disagree | | Disagree | | Slightly Disagree | | Slightly Agree | | Agree | | Strongly Agree | | | |
| | n | % | n | % | n | % | n | % | n | % | n | % | n | % |
| The instructor(s) had a good mastery of the curriculum. (n = 33,449) | 828 | 2.48 | 867 | 2.59 | 2,585 | 7.73 | 9,345 | 27.94 | 13,205 | 39.48 | 6,619 | 19.79 | 29,169 | 87.20 |
| The instructor(s) was well prepared for the lessons. (n = 33,433) | 719 | 2.15 | 789 | 2.36 | 2,183 | 6.53 | 8,567 | 25.62 | 13,381 | 40.02 | 7,794 | 23.31 | 29,742 | 88.96 |
| The instructor(s)' teaching skills were good. (n = 33,388) | 798 | 2.39 | 796 | 2.38 | 2,607 | 7.81 | 9,115 | 27.30 | 13,102 | 39.24 | 6,970 | 20.88 | 29,187 | 87.42 |
| The instructor(s) showed good professional attitudes. (n = 33,377) | 780 | 2.34 | 742 | 2.22 | 2,215 | 6.64 | 8,512 | 25.50 | 13,117 | 39.30 | 8,011 | 24.00 | 29,640 | 88.80 |
| The instructor(s) was very involved. (n = 33,387) | 705 | 2.11 | 677 | 2.03 | 2,043 | 6.12 | 8,178 | 24.49 | 13,103 | 39.25 | 8,681 | 26.00 | 29,962 | 89.74 |
| The instructor(s) encouraged students to participate in the activities. (n = 33,320) | 760 | 2.28 | 693 | 2.08 | 2,170 | 6.51 | 8,205 | 24.62 | 13,234 | 39.72 | 8,258 | 24.78 | 29,697 | 89.13 |
| The instructor(s) cared for the students. (n = 33,363) | 864 | 2.59 | 850 | 2.55 | 2,554 | 7.66 | 8,553 | 25.64 | 12,798 | 38.36 | 7,744 | 23.21 | 29,095 | 87.21 |
| The instructor(s) was ready to offer help to students when needed. (n = 33,355) | 815 | 2.44 | 723 | 2.17 | 2,159 | 6.47 | 8,254 | 24.75 | 13,189 | 39.54 | 8,215 | 24.63 | 29,658 | 88.92 |
| The instructor(s) had much interaction with the students. (n = 33,389) | 929 | 2.78 | 954 | 2.86 | 2,933 | 8.78 | 9,206 | 27.57 | 12,400 | 37.14 | 6,967 | 20.87 | 28,573 | 85.58 |
| Overall, I have a very positive evaluation of the instructors. (n = 33,444) | 923 | 2.76 | 739 | 2.21 | 1,982 | 5.93 | 7,809 | 23.35 | 13,226 | 39.55 | 8,765 | 26.21 | 29,800 | 89.10 |

respondents perceived that the instructors encouraged the students to participate. Third, as shown in Table 3, roughly four-fifths of the respondents perceived that the program promoted their development, including social competence (83.18%), emotional competence (82.45%), ability to resist harmful influences (83.84%), ability to distinguish between the good and the bad (85.35%), competence in making sensible and wise choices (84.02%), and overall development (84.36%). Fourth, while about four-fifths of the participants would recommend the program to their friends when they have similar needs, only a simple majority of them (69.02%) would join similar programs in the future (Table 4). Finally, more than four-fifths of the respondents indicated that they were satisfied with the program (Table 4). Regarding the degree of program adherence estimated by the workers, the mean level of adherence was 86.91%, with a range from 50 to 100%.

**TABLE 3**
**Perceived Effectiveness of the Program by the Program Participants**

| The extent to which the course (i.e., the program that all students have joined) has helped you: | 1 | | 2 | | 3 | | 4 | | 5 | | Participants with Positive Responses (Options 3–5) | |
|---|---|---|---|---|---|---|---|---|---|---|---|---|
| | Unhelpful | | Not Very Helpful | | Slightly Helpful | | Helpful | | Very Helpful | | | |
| | n | % | n | % | n | % | n | % | n | % | n | % |
| It has strengthened my bonding with teachers, classmates, and my family.(n = 33,546) | 1,962 | 5.85 | 5,261 | 15.68 | 13,105 | 39.07 | 10,022 | 29.88 | 3,196 | 9.53 | 26,323 | 78.47 |
| It has strengthened my resilience in adverse conditions. (n = 33,511) | 1,594 | 4.76 | 4,831 | 14.42 | 11,985 | 35.76 | 11,279 | 33.66 | 3,822 | 11.41 | 27,086 | 80.83 |
| It has enhanced my social competence. (n = 33,476) | 1,510 | 4.51 | 4,120 | 12.31 | 11,672 | 34.87 | 11,572 | 34.57 | 4,602 | 13.75 | 27,846 | 83.18 |
| It has improved my ability in handling and expressing my emotions. (n = 33,482) | 1,600 | 4.78 | 4,277 | 12.77 | 11,571 | 34.56 | 11,407 | 34.07 | 4,627 | 13.82 | 27,605 | 82.45 |
| It has enhanced my cognitive competence. (n = 33,454) | 1,628 | 4.87 | 4,345 | 12.99 | 11,550 | 34.53 | 11,302 | 33.78 | 4,629 | 13.84 | 27,481 | 82.15 |
| My ability to resist harmful influences has improved. (n = 33,442) | 1,519 | 4.54 | 3,886 | 11.62 | 11,187 | 33.45 | 11,550 | 34.54 | 5,300 | 15.85 | 28,037 | 83.84 |
| It has strengthened my ability to distinguish between the good and the bad. (n = 33,448) | 1,380 | 4.13 | 3,519 | 10.52 | 11,110 | 33.22 | 12,024 | 35.95 | 5,415 | 16.19 | 28,549 | 85.35 |
| It has increased my competence in making sensible and wise choices. (n = 33,433) | 1,438 | 4.30 | 3,906 | 11.68 | 11,193 | 33.48 | 11,733 | 35.09 | 5,163 | 15.44 | 28,089 | 84.02 |
| It has helped me to have life reflections. (n = 33,441) | 1,976 | 5.91 | 4,218 | 12.61 | 10,670 | 31.91 | 10,878 | 32.53 | 5,699 | 17.04 | 27,247 | 81.48 |
| It has reinforced my self-confidence. (n = 33,417) | 1,931 | 5.78 | 4,645 | 13.90 | 10,909 | 32.65 | 10,677 | 31.95 | 5,255 | 15.73 | 26,841 | 80.32 |
| It has increased my self-awareness. (n = 33,427) | 1,854 | 5.55 | 4,277 | 12.80 | 10,961 | 32.79 | 11,063 | 33.10 | 5,272 | 15.77 | 27,296 | 81.66 |
| It has helped me to face the future with a positive attitude. (n = 33,431) | 1,667 | 4.99 | 4,171 | 12.48 | 10,913 | 32.64 | 11,345 | 33.94 | 5,335 | 15.96 | 27,593 | 82.54 |
| It has helped me to cultivate compassion and caring about others. (n = 33,427) | 1,747 | 5.23 | 4,080 | 12.21 | 11,251 | 33.66 | 11,227 | 33.59 | 5,122 | 15.32 | 27,600 | 82.57 |
| It has encouraged me to care about the community. (n = 33,411) | 1,981 | 5.93 | 4,671 | 13.98 | 11,173 | 33.44 | 10,813 | 32.36 | 4,773 | 14.29 | 26,759 | 80.09 |
| It has promoted my sense of responsibility in serving the society. (n = 33,452) | 1,784 | 5.33 | 4,424 | 13.22 | 11,187 | 33.44 | 10,916 | 32.63 | 5,141 | 15.37 | 27,244 | 81.44 |
| It has enriched my overall development. (n = 33,451) | 1,609 | 4.81 | 3,624 | 10.83 | 10,228 | 30.58 | 11,442 | 34.21 | 6,548 | 19.57 | 28,218 | 84.36 |

Results based on several one-way between-subjects ANOVA indicated that there were no significant differences in the views of program (VP), views of workers (WK), and perceived program effectiveness (EF) across three school bandings (students with different levels of academic achievement). Results of independent-samples t-test also indicated that there were no significant differences in these three variables between schools adopting the 10-h core program mode and schools adopting the 20-h full program mode.

The means and standard deviations of VP, WK, and EF are shown in Table 5. As the VP and WK items used a 6-point Likert scale and the EF used a 5-point Likert scale, percentage score was computed for each of the variables in order to compare these three variables fairly. The formulas for the percentage scores are as follows: (1) VP100 = (VP-1)*100/5, (2) WK100 = (WK-1)*100/5, and (3) EF100 = (EF-1)*100/4. The means and standard deviations of the three percentage scores are given in Table 5. The results indicated that the WK100 had the highest mean (73.16) while the EF100 (60.33) had the lowest,

**TABLE 4**
**Other Aspects of Subjective Outcome Evaluation**

If your friends have needs and conditions similar to yours, will you suggest that they join this course? (n = 33,318)

| 1 | | 2 | | 3 | | 4 | | Participants with Positive Responses (Options 3–4) | |
|---|---|---|---|---|---|---|---|---|---|
| Definitely Will Not Suggest | | Will Not Suggest | | Will Suggest | | Definitely Will Suggest | | | |
| n | % | n | % | n | % | n | % | n | % |
| 2,053 | 6.16 | 4,412 | 13.24 | 20,889 | 62.70 | 5,964 | 17.90 | 26,853 | 80.60 |

Will you participate in similar courses again in the future? (n = 33,285)

| 1 | | 2 | | 3 | | 4 | | Participants with Positive Responses (Options 3–4)) | |
|---|---|---|---|---|---|---|---|---|---|
| Definitely Will Not Participate | | Will Not Participate | | Will Participate | | Definitely Will Participate | | | |
| n | % | n | % | n | % | n | % | n | % |
| 3,154 | 9.48 | 7,157 | 21.50 | 17,776 | 53.41 | 5,198 | 15.62 | 22,974 | 69.02 |

On the whole, are you satisfied with this course? (n = 33,257)

| 1 | | 2 | | 3 | | 4 | | 5 | | 6 | | Participants with Positive Responses (Options 4–6) | |
|---|---|---|---|---|---|---|---|---|---|---|---|---|---|
| Very Dissatisfied | | Moderately Dissatisfied | | Slightly Dissatisfied | | Satisfied | | Moderately Satisfied | | Very Satisfied | | | |
| n | % | n | % | n | % | n | % | n | % | n | % | n | % |
| 1,159 | 3.48 | 1,215 | 3.65 | 2,125 | 6.39 | 13,718 | 41.25 | 9,871 | 29.68 | 5,169 | 15.54 | 28,758 | 86.47 |

**TABLE 5**
**Means and Standard Deviations of the School Ratings Computed Based on Views of the Program Participants**

| Item | M | SD |
|---|---|---|
| Views of Program Participants (n = 213) | | |
| VP | 4.31 | 0.29 |
| VP100 | 66.28 | 5.75 |
| Views of the Workers Implementing the Program (n= 212) | | |
| WK | 4.66 | 0.28 |
| WK100 | 73.16 | 5.63 |
| Perceived Effectiveness of the Program (n= 213) | | |
| EF | 3.41 | 0.24 |
| EF100 | 60.33 | 6.09 |

*Note*: VP is the mean of the 10 items in Table 1, WK is the mean of the 10 items in Table 2, and EF is the mean of the 16 items in Table 3. Percentage scores: VP100 = (VP-1)*100/5, WK100= (WK-1)*100/5, and EF100 = (EF-1)*100/4.

with the VP100 (66.28) in the middle. Paired-samples t-tests were conducted on the following three pairs: (1) VP100 – WK100, t = –41.17 ($p < 0.001$); (2) VP100 – EF100, t = 27.11 ($p < 0.001$), and (3) WK100 – EF100, t = 42.61 ($p < 0.001$). In summary, all the pair comparisons were significant at $p < 0.001$, showing that there were significant differences involved even with Bonferroni correction.

## DISCUSSION

The present findings based on the subjective outcome evaluation strategy or client satisfaction survey showed that a high proportion of the respondents had positive perceptions of the program and the workers. Most importantly, roughly four-fifths of the respondents regarded the program as helpful to them. In short, the subjective outcome evaluation findings generally showed that the program participants had positive perceptions of the program as well as the workers who implemented the program. Nevertheless, it is noteworthy that although most of the participants had positive perceptions of the program, workers, and perceived effectiveness of the program, and about four-fifths of the participants indicated that they would recommend the program to peers with similar needs, only about 69% of them indicated that they would participate in similar programs in future. This observation is consistent with those reported previously[9,10], suggesting that there may be a dissociation between perception of a program and the behavioral intention to participate in a program. Another explanation is that the students might think that similar programs may have the same content, thus there is no need to join such programs again.

In the limited Western studies on the quality of program implementation, studies generally show that the degree of program adherence was not high. For example, Ringwalt et al.[17] found that one-fifth of the workers implementing the program did not use the curriculum guide at all and only 15% of them followed it very closely. Likewise, in their review of 12 schools implementing a school-based victimization prevention program, Melde et al.[18] found that only one-third of the schools delivered the program as intended and could be regarded as having high program fidelity. In contrast to the Western findings, the estimated level of program adherence was quite high in the present study. Actually, the mean estimated degree of adherence in the present study (86.9%) was highly comparable to the figures based on the ratings made by trained observers (co-walkers)[19,20]. It is conjectured that the training provided to the workers as well as the commitment of the workers (as reflected by the perceptions of the students) contributed to this high level of program adherence.

As far as the school ratings are concerned, results indicated that there were no significant differences in these ratings (VP, WK, and EF) by school bandings. In other words, the academic achievement of the students as indicated by the school banding (1 for the highest level of academic achievement, while 3 for the lowest level of academic achievement) indicated no differentiation in these ratings. This is possibly due to the fact that the Project P.A.T.H.S. is not an academically oriented program, but a comprehensive, positive development or whole-person education program. The practical implication of this finding is that students who are less capable in academic domains may also enjoy and benefit from generic programs that deal with their all-round psychosocial development.

Previous research studies showed that well-evaluated programs should have adequate duration[1]. The present results indicated that there were no significant differences in the three school ratings (VP, WK, and EF) by mode of implementation (Group 1: 10 h; Group 2: 20 h). While the evaluation by the program participants did not differ in these two different implementation modes, future studies should investigate whether the intervention effect is linearly related to program duration. As aggregated data based on schools instead of individuals were used to test this hypothesis, it would be desirable to use individual scores as the basic unit of analyses.

The present study also showed that the mean of WK100 was the highest and that of EF100 was the lowest, with VP100 in the middle. The results indicated that participants gave significantly higher ratings to items concerning the workers (teachers and social workers) who implemented the program (WK) than to items concerning the curriculum design and classroom atmosphere (VP), as well as the effectiveness of the program (EF). In other words, the instructors were in general appraised the highest, whereas the perceived effectiveness is relatively less favorably evaluated, with the general feature of the program including the classroom atmosphere in the middle. This finding appears to be quite natural and explicable because EF items may appear a bit difficult to some program participants because some of the items appear to be quite abstract and some of them concern long-term effects. On the other hand, program participants know their instructors and perhaps have built up a positive relationship with the program instructors. In general, the findings imply that the success of the program relies much on the quality of teaching provided by the teachers and social workers who implement the program. Nevertheless, it is noteworthy that the mean scores in these three domains can be regarded as on the high side in terms of the absolute values of the scores.

The present subjective outcome evaluation findings are important because they are based on a large sample size (n = 33,867 students involving 213 schools) and correlates of the subjective outcome evaluation ratings are rarely examined in the literature. Nevertheless, several limitations of the present study should be highlighted. First, as the data were reconstructed from the reports submitted by the schools, the unit of analysis was schools rather than individual program participants, which substantially reduces the power of statistical analyses. Second, while the present findings could be interpreted in terms of positive program effects and experiences of the program participants, it should be noted that there are several alternative explanations, such as demand characteristics (which was not high in the present study though). Despite these limitations, the present findings suggest that the Tier 1 Program and its implementation were perceived in a positive manner by the program participants and they perceived the program to be beneficial to their own development. These positive findings are important because they suggest that the program can successfully engage the students in the program implementation process. From a program evaluation point of view, as systematic evaluation of social services is at its infancy in different Chinese contexts, the present paper constitutes a model based on which future subjective outcome evaluation studies can be conducted[21,22].

## ACKNOWLEDGMENTS

The preparation for this paper and the Project P.A.T.H.S. were financially supported by The Hong Kong Jockey Club Charities Trust.
